# A narrative review on the role of cytokines in the pathogenesis and treatment of familial Mediterranean fever: an emphasis on pediatric cases

**DOI:** 10.3389/fped.2024.1421353

**Published:** 2024-07-26

**Authors:** Ahlam Chaaban, Hasan Yassine, Razane Hammoud, Ruba Kanaan, Louna Karam, José-Noel Ibrahim

**Affiliations:** Department of Natural Sciences, School of Arts and Sciences, Lebanese American University (LAU), Beirut, Lebanon

**Keywords:** familial mediaterranean fever, children, cytokine, treatment, pathogenesis

## Abstract

Familial Mediterranean Fever (FMF) is a hereditary autoinflammatory disease characterized by an early onset of recurrent fever and serositis episodes. FMF is caused by mutations in the *MEFV* gene which encodes the pyrin protein, an IL-1β mediated inflammation regulator. Recent findings have identified a plethora of molecules and pathways involved in the regulation of inflammation and innate immunity, hence increasing our understanding of the etiology and inflammatory nature of FMF. Cytokines, in particular, have been found to play a key role in the pathogenesis and treatment of the disease. Indeed, various studies associated cytokines’ genetic variations and expression with susceptibility to and severity of the disease, which was further supported by the positive response of patients, both children and adults, to targeted cytokine blocking therapies. These studies highlighted the potential use of cytokines as biomarkers and target in resistant/intolerant patients and contributed to improving the early detection of FMF in children, thus enhancing their quality of life and providing alternative treatment for severe cases. The aim of this review is to provide the latest updates on the pivotal role of cytokines in FMF and to discuss the efficacy and safety of anti-cytokine biologics by primarily focusing on pediatric FMF cases.

## 1 Introduction

The dysregulation of the immune system produces an array of diseases that include but are not limited to autoimmune and autoinflammatory disorders. On one hand, autoimmune diseases (AIDs) are mainly mediated by autoantibodies or autoreactive T cells, leading the adaptive immune system to attack the body's own cells, causing chronic inflammation and tissue destruction (1). On the other hand, AIDs encompass a heterogenous group of conditions that often involve mutations in genes related to cytokines and inflammasomes, protein complexes that activate the innate immune system (1). Within the broad spectrum of AIDs, monogenic AIDs (mAIDs) are a group of rare genetic disorders often beginning in childhood. These diseases arise from mutations in a single gene linked to the innate immune system, leading to a systemic inflammation accompanied by an overproduction of pro-inflammatory cytokines and acute phase reactants (2).

Familial Mediterranean Fever (FMF) is the earliest described and most frequently encountered mAID (1). It affects people living in or originating from the Mediterranean area, mainly Jews, Armenians, Turks, and Arabs (3). Indeed, Turks are considered to have the highest prevalence followed by Armenia with estimated rates of 1:150–1:1,000 and 1:400–1:500 respectively (3, 4). FMF typically presents in early childhood, with rare late-onset disease manifestations (5). It is characterized by recurrent bouts of fever which develop before 10 and 20 years of age in 75% and 90% of the cases, respectively (6). Hence, the disease may be represented as a form of pediatric hereditary recurrent fever (HRF), with no reported prevalence of FMF in children yet. While fever, abdominal, and joint pain remain the most common symptoms, the clinical presentation of FMF in children appears to be broader than previously discussed. Thoracic pain, skin rash, and even scrotal swelling were also found to be manifested in children, with some presenting solely with fever, making the diagnosis more challenging (7).

FMF is caused by mutations in the *MEFV (MEditerranean FeVer)* gene which is located on the short arm of chromosome 16 (16p13.3) and is composed of 10 exons (8, 9). The typical mode of inheritance of FMF is autosomal recessive; however, FMF cases with a single *MEFV* mutation or a dominant transmission are increasingly reported (10). To date, 62 pathogenic/likely pathogenic variations are identified (11). The M694V, V726A, M680I, and M694I mutations, located in exon 10, and the E148Q variant, found in exon 2, account for approximately 85% of FMF cases (12). While the frequency of these variants differs largely between countries and populations, the M694V is reported to be the most common mutation among FMF patients and is associated with the most severe phenotypic expression of the disease (13). In addition to genetics, epigenetic factors play a major role in FMF manifestations making it a complex disease (14).

**Figure 1:**
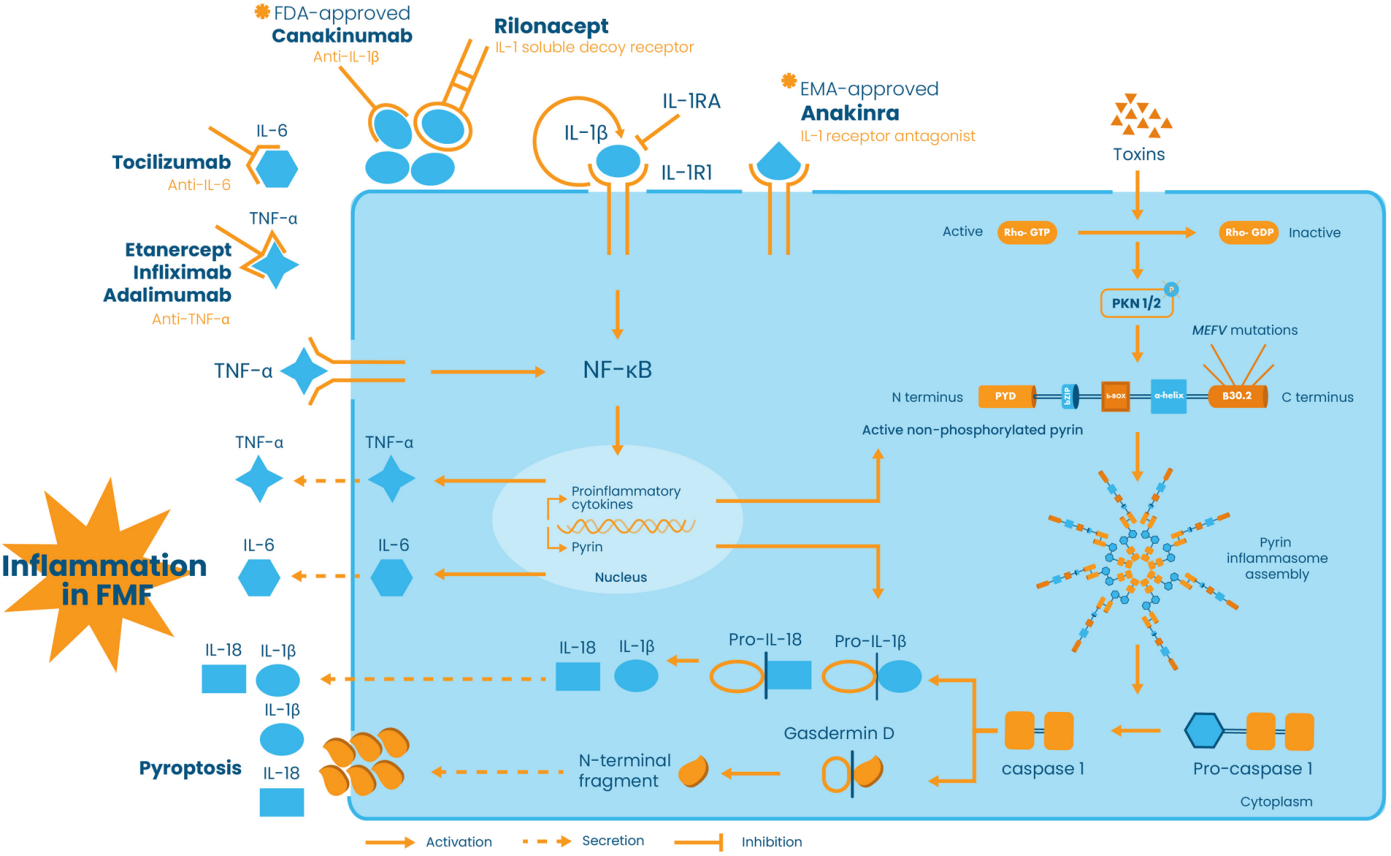
Schematic representation of pyrin inflammasome activation and the role of cytokine network and anti-cytokine therapies in FMF pathogenesis and treatment. In the presence of stimuli, such as bacterial toxins, Rho-GTP is converted into its inactive form Rho-GDP, thus preventing the activation of the signaling molecules protein kinase N1 (PKN1) and protein kinase N2 (PKN2), phosphorylating pyrin. In addition to toxins, pyrin can be activated by *MEFV* mutations. The active non-phosphorylated or mutated form of pyrin induces pyrin inflammasome assembly and subsequently the autocatalytic activation of pro-caspase-1 into caspase-1. Caspase-1 in turn converts pro-IL-1β and pro-IL-18 into their active IL-1β and IL-18 forms and cleaves Gasdermin D responsible for membrane pores formation and pyroptosis. IL-1β is secreted into the extracellular space and binds to its receptor IL-1R1, hence activating the NF-κB signaling pathway and promoting its own expression, via a positive feedback loop, as well as the expression of other pro-inflammatory cytokines, namely, IL-6, TNF-α, and IL-18. On the other hand, TNF-α is able to modulate pyrin expression and to promote pyrin inflammasome activation. Extracellularly, all these molecules create a “cytokine storm” that leads to the inflammatory phenotype of FMF. Pro-inflammatory cytokines are targeted via antagonists or antibodies. Anakinra, approved by the European Medicines Agency (EMA), is an IL-1β antagonist which competes with IL-1β for the binding of IL-1R1, whereas rilonacept and canakinumab, approved by the Food and Drug Administration (FDA), are antibodies binding to IL-1β and preventing its binding to IL-1R1. On the other hand, IL-6 is targeted by anti-IL-6 tocilizumab and TNF-α is neutralized by anti-TNF-α, namely, etanercept, infliximab, and adalimumab.

Mutations in the *MEFV* gene orchestrate abnormal inflammation in FMF via a 95 kDa protein named pyrin or marenostrin. Pyrin is composed of 781 amino acids and is mainly found in immune cells like granulocytes, eosinophils, monocytes, and dendritic cells. It is composed of five different domains: the N-terminal pyrin domain (PYD), bZIP transcription factor domain, B-box zinc finger domain, α-helical coiled-coil domain, and the C-terminal B30.2 domain. The latter domain plays the most important role in FMF pathogenesis since most of the disease-penetrant *MEFV* mutations cluster in this region (14). Pyrin activation depends on the detection of homeostasis-altering molecular processes, defined as perturbations in cytoplasmic homeostasis, and on RhoA inactivation (15). Indeed, in physiological conditions, the RhoA protein activates signaling molecules protein kinase N1 (PKN1) and protein kinase N2 (PKN2), phosphorylating pyrin. This modification allows pyrin to interact with 14-3-3 proteins preventing it from inducing inflammasome assembly. As a result, the pyrin inflammasome remains inactive, and the cell maintains a balanced inflammatory state (16, 17). However, in FMF, mutated active pyrin is unable to interact with the 14-3-3 inhibitory proteins. Consequently, the abnormal pyrin binds to ASC (Apoptosis-associated speck-like protein containing a CARD) protein, a key component of the inflammasome, resulting in the auto-catalytic activation of caspase-1. Caspase-1, in turn, cleaves pro-interleukin-1 beta (pro-IL-1β) and pro-IL-18 into their active forms, IL-1β and IL-18, as well as Gasdermin D (GSDMD) which is primarily responsible for pyroptosis. GSDMD forms membrane pores that promote the release of the mature forms of IL-1β and IL-18 (16–18), hence stimulating the expression of genes involved in the IL-1 inflammatory pathway, inducing a positive feedback loop that further amplifies inflammation, contributing to FMF’s recurrent inflammatory attacks (16, 17) (Figure 1).

The role of IL-1β in FMF pathogenesis extends beyond its initial release. Its activation acts as a signaling flare, triggering the production of other pro-inflammatory cytokines by various immune cells (19). Key players in this response include but are not limited to TNF-α, IL-6, IFN-γ, and IL-17 (20). On one hand, pro-inflammatory cytokines join forces with IL-1β, creating a potent “cytokine storm” that exacerbates the inflammatory response (21). On the other hand, anti-inflammatory cytokines, such as IL-4 and IL-10, exert negative feedback to control the pro-inflammatory cytokine response (22). The balance between pro- and anti-inflammatory cytokines defines the severity of the inflammation and the overall disease’s manifestation; any dysregulation in the inflammatory response might result in the progression of the disease and the development of comorbidities such as hearing loss (23), inflammatory bowel disease (24), and cryptogenic cirrhosis, especially among children (25). In this respect, a large number of studies focused on studying the cytokine profile in FMF and on establishing possible associations of cytokines polymorphisms and expression levels with disease susceptibility and/or severity. These reports led to the identification of different modifying genes of the disease and offered interesting insights into potential therapeutic targets and approaches to manage the disease and its progression (20, 26). Although the first line of defense against FMF attacks is a daily dose of colchicine, hindering neutrophil activation and disrupting microtubule formation (27, 28), it does not always provide complete control due to resistance or intolerance in some FMF patients (29, 30). In addition, colchicine has usage limitations due to its narrow therapeutic index and potential side effects such as gastrointestinal disturbances and nausea (31). For this reason, biological drugs that target specific cytokines, namely, IL-1, IL-6, and TNF-α are prescribed in colchicine intolerant/resistant patients. The EMA (European Medicines Agency) and FDA (Food and Drug Administration) approved IL-1 inhibitors, anakinra and canakinumab, have been shown to be safe and effective in reducing the frequency and intensity of FMF attacks, and potentially preventing amyloidosis, the most severe complication of FMF (32–34) (Figure 1).

Evidently, studying the role of cytokines in FMF pathogenesis has been of clinical significance. However, despite the relevance and importance of these studies, there remains much to be elucidated, especially when it comes to children. Accordingly, this review aims to provide an updated overview on the involvement of cytokines in the pathogenesis and treatment of FMF. We will present on one hand research exploring cytokine polymorphisms and expressions in FMF and their association with susceptibility to and/or severity of the disease and, on the other hand, studies evaluating anti-cytokine therapies in the management of FMF, particularly among children. We will also address throughout the document the limitations of these studies and identify areas where further investigation is needed. This knowledge is instrumental for identifying potential diagnostic and prognostic biomarkers and for developing effective treatment strategies, especially at younger ages.

## 2 Methodology

This narrative review aims to provide an updated overview on the role of cytokines in FMF pathogenesis and treatment with an emphasis on pediatric cases (age below 18). To achieve this aim, a comprehensive methodology was adopted. Literature search was conducted using databases such as PubMed, Google Scholar, Scopus, and Medline with the following keywords: “Familial Mediterranean Fever” or “FMF” or “pediatric FMF” or “FMF children” and “cytokines” or “cytokine polymorphisms” or “cytokine expression” or “anti-cytokine therapy” or “anti-IL-1 biologics”. In addition, related articles were identified by examining some review articles and their reference lists.

Our review included studies published in the English language. Given the minimal number of studies investigating the association of cytokine gene polymorphisms with FMF, we included all published and relevant findings to reflect their potential implications on pediatric FMF patients. As for cytokine expression in FMF, research on children was limited; therefore, we developed studies conducted in the general FMF population, mainly those documented after the last review on cytokine signature and profiles in HRFs (35). In regard to the therapeutic use of cytokines in FMF, literature was extensive; hence, only published data in the last 10–15 years on children was included, with the exception of two studies conducted on adults (anti-TNF-α and anti-IL-6 therapeutics) (36, 37) due to the lack of data on pediatric cases. The screening process was initially based on title and abstract revision, followed by full-text overview to validate the relevance of the studies. The retrieved data involved study design, demographic characteristics, main findings, cytokine roles, and treatment responses. The review summarized and interpreted the selected studies and highlighted some gaps while suggesting future research directions.

## 3 Cytokine profile and signature in FMF

Cytokines are peptides, proteins, or glycoproteins that are involved in immune cell communication (38). They play a role in regulating immune tolerance by stimulating the activation and differentiation of cells involved in orchestrating an immune response (39). In fact, there are more than 120 “essential cytokines”, cytokines that are linked to at least one disease, identified (40). Due to the large number of cytokines that has been discovered, there has been a need to classify “essential cytokines” based on their presumed function, mode of action, and cell of secretion. In terms of function, cytokines could mitigate immune responses through two opposing mechanisms further classifying them into pro-inflammatory and anti-inflammatory cytokines (41). Today, the term “cytokine” encompasses a broad category of factors that are secreted by immune cells and/or acting on immune cells; it includes interleukins, lymphokines, monokines, interferons, colony stimulating factors, mesenchymal growth factors, and chemokines (42).

As aforementioned, cytokine dysregulation could lead to a myriad of diseases due to the role they play in immune responses (43). In the context of HRFs, cytokines trigger systemic inflammation in the absence of infectious stimuli (44). In order to better understand the role of the cytokine network and its pathophysiological implication in HRFs, our research team conducted, in 2017, a review of the data published on cytokines’ profiles in HRFs at the serum, *ex vivo*, and transcript levels (35). Interestingly, the review integrated data in an updated classification of HRFs in which FMF was classified as an inflammasomopathy. The activation of the cytokine network has been related to mutations across pyrin which triggers the inflammasome cascade inducing a dysregulation in cytokine levels (26). Indeed, at the serum level, pro-inflammatory cytokines IL-6, IL-12, IL-17, IL-18, soluble IL-2 receptor (sIL-2R), interferon-gamma (IFN-γ), and tumor necrosis factor alpha (TNF-α) were reported to be higher in FMF patients during or between attacks than in healthy controls, supporting the ongoing subclinical inflammation in FMF (35). As for the anti-inflammatory cytokine IL-10, contradictory results between populations were portrayed and no clear-cut cytokine pattern has been yielded. At the transcriptional level, IL-6, TNF-α, and IL-1β showed higher expression levels in FMF patients during attack-free period compared to healthy controls. More importantly, the review highlighted the relevance of *ex vivo* approaches in defining cytokine signatures for the different HRFs and their use as biomarkers to improve the diagnosis, management, and treatment of these rare diseases. In fact, since measurement of circulating cytokine levels has not been a reliable indicator of their fundamental role in AIDs, mostly because cytokines are very labile and some are subject to rapid degradation, alternative approaches have been designed to assess cytokine levels in supernatants of resting and activated PBMCs (peripheral blood mononuclear cells) and neutrophils cultured *ex vivo*. For instance, our study revealed that unlike other HRFs, mainly CAPS (Cryopyrin-associated periodic syndromes) and NLRP12AD (NLRP12-associated disorders), pyrin inflammasome is not constitutively activated in FMF. Moreover, we were able to define a specific cytokine “signature” to FMF patients characterized by increased levels of Th17 cytokines and diminished levels of Th1 and Th2 cytokines. Finally, our findings revealed a correlation between levels of inflammatory cytokines, namely, IL-1β, IL-1α, IL-6, IL-17, and IL-22 and penetrance of *MEFV* mutations; cytokine production was increased in M694V homozygous FMF patients when compared to those heterozygous for the same mutation or with other genotypes, suggesting that the wide clinical variability observed in FMF patients is partly related to the penetrance of *MEFV* mutations and allelic heterogeneity (20, 35).

Despite the relevance of the data presented in the review, it did not cover the studies associating cytokine polymorphisms with susceptibility to and severity of FMF. Moreover, it did not explore in-depth the efficacy and safety of anti-cytokine therapies, in particular among FMF children. Finally, during the last years, numerous studies explored the involvement of a wider range of cytokines in the pathogenesis of FMF; these include, but are not limited to, IL-4, IL-18, macrophage migration inhibitory factor (MIF), vascular endothelial growth factor (VEGF), chemokines, and the S100A protein family (45–51).

### 3.1 IL-1β and IL-1RA

IL-1 family cytokines are generally secreted by macrophages, large granular lymphocytes, B cells, endothelium, fibroblasts, and astrocytes with B cells, macrophages, endothelium, and tissue cells being the primary target (52). The role of pyrin inflammasome and IL-1β has been well-established in pediatric AIDs, especially FMF (15). Recently, it was suggested that the mechanism by which pyrin inflammasome induces FMF is through an inherently reduced interleukin-1 receptor antagonist (IL-1RA) expression specific to pyrin inflammasome which suggests a diminished anti-inflammatory capacity leaving the patients susceptible to pro-inflammatory stimuli, irrespective of colchicine therapy (53). In this respect, the genetic variability in *IL-1β* and *IL-1RA* genes and their expression levels have been extensively studied in adult FMF patients but not in children. For instance, a recent study conducted by Yigit et al. compared the distribution of the rs2234663 *IL-1RA* 86 bp *VNTR* (variable number of tandem repeats) between 172 Turkish FMF patients, of whom 62 had amyloidosis, and 120 healthy controls. The *IL-1RA* VNTR A1/A2 and A1/A4 genotypes and A1-A4 alleles were more common in both patient groups than controls suggesting that the A1-A4 alleles are associated with FMF but not with amyloidosis in FMF patients (54). Nevertheless, these findings are contradictory to those obtained in another study conducted on 160 FMF patients where *IL-1RA* VNTR was not associated with FMF risk in the Turkish population (55). Similarly, a research conducted on 42 Lebanese FMF patients revealed no significant difference in the distribution of the *IL-1RA* VNTR genotypes and alleles between patients and controls (26). Differences in findings might be attributed to the study design, sample size, and patients’ characteristics. In the latter study, the effect of three polymorphisms in *IL-1β* gene, rs16944 *IL-1β* (−511C/T), rs1143627 *IL-1β* (−31 T/C), and rs1143634 *IL-1β* (+3,954 T/C), on the severity and occurrence of FMF was also assessed (26). Interestingly, the CC genotype and C allele at positions −31 and + 3,954 of *IL-1β* gene were more frequent in FMF patients than in controls, hence being associated with an increased risk of the disease. Moreover, patients carrying the *IL-1β* −31 CC genotype had a significant increase in LPS-induced IL-1β secretion and a more severe clinical presentation of the disease when compared to patients carrying the TC and TT genotypes, playing therefore a role in FMF severity. These findings provided evidence of the role of *IL-1β* as an *MEFV*-independent modifier gene for FMF.

Several additional studies further assessed the expression levels of IL-1β in FMF where the cytokine has been found to be significantly overexpressed in patients’ polymorphonuclear neutrophils (PMNs) and serum compared to controls (56–58). In one study, Martirosyan et al. sought to test whether cytoskeletal dynamics in the absence of pathogens may cause an abnormal activation of PMNs in FMF patients by quantifying IL-1β production in supernatant of cells cultured *ex vivo* (56). The study included 35 patients, 21 attack-free patients and 14 patients during attack, and 20 age- and gender- matched healthy subjects. Findings revealed a spontaneous and induced activation of patients’ neutrophils after transmigration as seen by the increased expression of IL-1β, thus highlighting a heightened pathogen-independent sensitivity of mutated pyrin inflammasome to cytoskeletal modifications. IL-1β was assessed in another research, along with IL-18 and caspase-1, in the serum of 60 FMF patients (30 in attack and 30 free of attacks) and 30 healthy controls using ELISA with the main purpose to investigate the possible relationship of these markers with disease severity and acute phase reactants in FMF (57). When compared to the control group, all three markers were increased during attacks, but only caspase-1 levels were significantly higher in patients in attack-free period. Moreover, a positive correlation was detected between IL-1β levels and disease severity scores as well as acute-phase reactants levels during attacks. Accordingly, the authors suggested the use of IL-1β, IL-18, and caspase-1 as disease activity markers; caspase-1 could also be beneficial during attack-free period as a diagnostic tool. Finally, in order to better understand the role of RAC1, a member of the Rho family in FMF inflammatory pathogenesis, our research team evaluated *RAC1* gene expression levels and the effect of RAC1 inhibition on *ex vivo* IL-1β and caspase-1 production as well as oxidative stress generation in FMF (58). *RAC1* expression and LPS-induced IL-1β and caspase-1 levels were increased in PBMC supernatants of patients in attacks compared to those in attack-free period or controls. In addition, LPS-stimulated neutrophils of FMF patients secreted higher amounts of the oxidative stress marker malondialdehyde (MDA) compared to controls, while levels of the antioxidant systems, catalase and reduced glutathione (GSH), were reduced in unstimulated neutrophil cultures. Interestingly, RAC1 inhibition resulted in a decrease in IL-1β, caspase-1, and MDA levels and an increase in catalase and GSH activities. In light of these findings, we concluded that RAC1 may exercise its role in the FMF inflammatory process, either through mediation of caspase-1-dependent IL-1β secretion and/or via alteration of the redox homeostasis leading to oxidative stress generation.

### 3.2 IL-18

Given the relevance of IL-18 in the pyrin inflammasome, it was not surprising that extensive research has been recently conducted to decipher its role in FMF pathogenesis. For instance, Koga and colleagues aimed to identify the utility of the measurement of multiple cytokines including a specific combination of biomarkers for clinical application (59). IL-18, along with 44 other cytokines, was analyzed using mutlisuspension cytokine array in serum of 75 FMF patients and 40 age-matched controls. IL-18 levels were significantly higher in FMF attack group compared to patients in attack-free period and to healthy controls. More importantly, using multivariate classification algorithms and a logistic regression analysis, it was shown that the combined quantification of IL-18, IL-17, and IL-6 was the best to distinguish FMF patients in attack from the healthy population. In order to characterize the longitudinal course of circulating IL-18 in FMF patients after treatment initiation, another research team performed serial analysis of serum IL-18 in 12 FMF patients carrying mutations in exon 10 of *MEFV* gene. After a follow-up of more than 4 years, authors were able to identify two subgroups based on changes in serum IL-18 over time: group A, including 7 patients, in which IL-18 levels declined progressively after colchicine treatment and group B consisting of 5 patients showing continued elevation of circulating IL-18, despite declines in IL-6. These findings support the previously reported data by Gohar et al. (60) who concluded that IL-18 is highly sensitive in detecting subclinical inflammation in patients with FMF and suggest that longitudinal IL-18 quantification may contribute to better follow-up of FMF patients (61). More recently, Stoler et al. analyzed inflammatory responses in FMF and characterized gene–dose effects at the cellular level, by comparing *ex vivo* IL-18 production and neutrophil activity using reverse transcription-quantitative polymerase chain reaction (RT-qPCR) in 12 patients with FMF, 6 patients with diverse inflammatory diseases, and 9 controls (62). Spontaneous IL-18 release was found to be the highest in patients with two *MEFV* mutations, followed by healthy heterozygous *MEFV* mutation carriers who also exhibited greater IL-18 levels as compared to controls. On the other hand, blocking IL-1β did not abolish the secretory potential of neutrophil, hence indicating that neutrophil activation is independent from IL-1 activation and displays a gene–dose effect responsible for genotype-dependent phenotypes. Another *ex vivo* study aimed to evaluate the effect of *MEFV* mutations on the ability of pyrin to detect inflammasome stimuli including RhoGTPase inhibition. In this respect, IL-18 and IL-1β production was assessed using ELISA in monocytes of 48 FMF patients and 26 healthy donors upon stimulation with a pyrin-activating stimulus, *Clostridium difficile* toxin B (TcdB) (63). Stimulated monocytes secreted more IL-18 and IL-1β than controls. In addition, in line with the findings of Stoler and colleagues, monocytes from patients carrying two *MEFV* mutations displayed an increased pyrin inflammasome response compared to monocytes from patients with a single pathogenic variant. *MEFV* mutations were also found to decrease the activation threshold of pyrin inflammasome which might contribute to the increased release of IL-18 and IL-1β in FMF patients. Finally, it is noteworthy to mention that despite the significant number of studies targeting FMF adults, to the best of our knowledge, no studies have assessed the expression of IL-18 in children. Moreover, no studies explored the effect of *IL-18* genetic variations on IL-18 production and pyrin inflammasome activation.

### 3.3 IL-6

IL-6 is synthesized by T and B lymphocytes, fibroblasts, and macrophages with the primary effects being B-cell differentiation and acute phase protein production (64). As mentioned previously, IL-6 has been thoroughly studied in FMF, particularly in adults, and serum and *ex vivo* levels were found to be increased in patients as compared to controls (35, 59, 65). Moreover, it was reported that the combined measurement of IL-6, granulocyte colony stimulating factor (G-CSF), IL-10, and IL-12p40 is a helpful marker in differentiating attack periods from attack-free periods (59). In the context of children, no studies assessed IL-6 production in serum or supernatants of patients; however, a study investigating the relationship between rs1800795 *IL-6* (−174G/C) polymorphism and the clinical features, disease severity score, and proteinuria has been conducted in 99 children with FMF in comparison to 157 controls (50). Of these 99 patients, 26 children were homozygous for mutations in *MEFV*, 40 were heterozygous, and the remaining 33 were compound heterozygous. The study found no statistically significant difference with regard to the distribution of the *IL-6* (−174G/C) genotype and allele frequencies between patients and controls. Furthermore, no direct association was detected between the tested polymorphism and the frequency of FMF clinical features and disease severity score. Likewise, a study assessed the potential association between *IL-6* (−174G/C) polymorphism and amyloidosis in 156 FMF adult patients, of whom 80 had amyloidosis. Data showed no significant difference in genotype and allele frequencies between patients and controls. Additionally, no association was detected between the tested polymorphism and risk of amyloidosis (66). In this respect, the authors concluded that the increased production of IL-6 during and between attacks is not related to this particular variation in the *IL-6* gene but rather to the inflammatory nature of the disease. It would be interesting in future studies to investigate additional genetic variations in *IL-6* and to correlate them with IL-6 production and subsequently disease severity in children with FMF.

### 3.4 TNF-α

TNF-α is secreted by monocytes, activated macrophages, and natural killer cells. It is involved in recruiting other cytokines and chemokines, regulating inflammation, and inducing cell death (67). The role of TNF-α in the pathogenesis of FMF has been well-established where it has been found to be a critical modulator of pyrin expression, inflammation, and pyrin-inflammasomopathy (68). Levels were also reported to be increased in attack and attack-free patients compared to controls (35, 69). Besides that, TNF-α has been considered as a potential differentiating biomarker between FMF and sepsis when its levels were co-determined with granulocyte-monocyte colony-stimulating factor (GM-CSF) (65). As such, several studies sought out to explore the association of different polymorphisms in *TNF-α* gene with the cytokine’s production and disease outcome. The first research conducted in 2003 in Turkey assessed whether the rs1800629 *TNF-α* (−308G/A) and *SAA1.1* polymorphisms play a role in progression of amyloidosis in FMF patients. The patient group included 126 unrelated FMF patients, of whom 45 had amyloidosis, and a control group consisting of 79 healthy individuals. Findings revealed no significant difference in the distribution of the *TNF-α* −308A allele across the three different studied groups, hence suggesting no association between this polymorphism and occurrence of FMF or amyloidosis (70). This research was followed by another one investigating the involvement of *TNF-*α (−308G/A) with another polymorphism, rs361525 *TNF-*α (−238G/A), in FMF. To note, these polymorphisms, located in the promoter of *TNF-*α gene, were reported to play a role in TNF-α increased production (71, 72). The distribution of genotype and allele frequencies of the tested polymorphisms were found to be comparable in the patients’ and controls’ groups. Furthermore, there was no significant association between *TNF-*α (−238G/A) and *TNF-*α (−308G/A) genotypes and the frequency of attacks in FMF. Therefore, the authors concluded that both polymorphisms do not appear as major genetic risk factors for the susceptibility to FMF or the severity of the disease (73). In 2012, Bonyadi et al. aimed to evaluate the role of rs1799964 *TNF-*α (−1,031 T/C) and *TNF-*α (−308G/A) polymorphisms in 86 patients carrying the M694V homozygous mutation in comparison to 100 matched healthy controls from Iran. Similar to previous reports, both polymorphisms showed no significant difference in terms of genotype and allele distribution between patients and controls. Interestingly, although the analysis of *TNF-*α (−1,031 T/C) polymorphism did not reveal a significant correlation with the manifestation and progression of clinical characteristics of FMF, a statistically significant difference was observed between patients with and without arthritis or amyloidosis with regard to *TNF-*α (−308G/A). These results suggest that individuals with the *TNF-*α −308 GG genotype may exhibit an increased susceptibility to amyloidosis and arthritis, whereas the *TNF-*α −308 A allele may have a protective role among this cohort of FMF patients carrying the M694V/M694V genotype (74). To summarize, further investigations in other populations are needed to reach a conclusion on the lack of association between *TNF-*α polymorphisms and FMF.

### 3.5 IL-4

IL-4, a potent regulator of immunity, is synthesized primarily by Th2 cells, mast cells, eosinophils, and basophils (75). It plays a major role in Th2 cell-mediated immunity, IgE and IgG1 class switching in B cells (76), and alternative macrophage activation (77). Additionally, IL-4 has been found to mediate regulatory T cells (T-reg) immunosuppression (78). In this regard, assessing IL-4 levels and the role of genetic variations in *IL-4* gene with FMF has been a topic of interest. In a study of 50 Egyptian children with FMF (25 in acute FMF attack and 25 in attack-free period), the rs79071878 polymorphism, a 70 bp VNTR located in intron 3 of *IL-4*, was assessed and compared to 40 age- and gender- matched controls (49). The study showed no significant difference in terms of genotype or allele distribution between patients and controls and no correlation with FMF severity or response to colchicine therapy. Moreover, IL-4 levels were comparable in serum of patients, during attacks or in attack-free periods, and controls. Authors also found no correlation with participants’ demographics or clinical characteristics, disease severity, or response to colchicine therapy. Nevertheless, when the same polymorphism was assessed in 160 adults with FMF in Turkey compared to 120 controls, the results showed a significantly higher frequency of P1P1 genotype in patients than in controls (55), but no significant difference was found with regard to the allelic frequency of the *IL-4* VNTR polymorphism. Two other Turkish studies assessed the role of the same polymorphism in FMF susceptibility by comparing genotype and allele frequencies between 339 FMF patients and 331 healthy controls. The researchers found that the P1 allele and P1P1 genotype were associated with an increased risk of FMF; however, a non-significant correlation was found between IL-4 blood serum levels and the pathogenesis of the disease (79). Within the same population, 62 FMF patients with amyloidosis and 110 FMF patients without amyloidosis were compared to 120 controls. The results showed that the *IL-4* VNTR P1 allele was more common in FMF patients with amyloidosis compared to controls, but no significant difference was present between the groups of patients (54). Discrepancy in findings between these studies might be attributed to differences in the sample’s size and demographics such as gender, age, race, and ethnicities.

### 3.6 MIF

In recent years, it has been shown that MIF is produced by T-cells, macrophages/monocytes, eosinophils, PMNs, and epithelial cells, thus exhibiting a pleiotropic activity (80). MIF has been found to play a major role in regulating the immunosuppressive effects of glucocorticoids (81). In addition, MIF exhibits several immunological and hormonal functions that include increasing the expression of pro-inflammatory cytokines, adhesion molecules, and chemokines (81–83). It is also involved in sustaining immune cell survival by inhibiting apoptosis through stimulating inflammatory responses (84). Therefore, MIF functions as an immune-regulating protein in both the innate and adaptive immune responses and its levels may reflect the inflammatory state and activity of FMF. Accordingly, many researchers found it interesting to assess the contribution of MIF to FMF pathogenesis. Interestingly, a study evaluating the expression levels of MIF protein by ELISA in serum of 51 patients and 30 healthy controls showed that MIF levels were significantly higher in patients than in controls (46). In order to understand the reason underlying MIF overexpression in FMF patients, another research team investigated the potential association between rs755622 (−173G/C) polymorphism in *MIF* and disease susceptibility in 98 children with FMF compared to 157 healthy subjects. Findings revealed that individuals with the CC allele seem to have a higher predisposition to FMF; however, there was no significant difference between patients and controls in terms of allele frequency (85). These findings were very recently supported by Yigit and colleagues who reported a significant association of rs755622 *MIF* (−173G/C) with FMF susceptibility, but not with the risk for amyloidosis (51). It is noteworthy to mention that the functional tetranucleotide CATT repeat at position −794 of the *MIF* gene (rs8544572) represents another interesting polymorphism to study in FMF. Indeed, it has been reported that MIF expression increase, which is positively correlated to the number of CATT repeats and the activity of the *MIF* promoter, is associated with an enhanced severity and risk for rheumatoid arthritis (86).

### 3.7 VEGF

Due to the inflammatory nature of FMF, patients tend to experience high risks of cardiovascular events (87). The manifestation of these events is modulated by different molecules including the cytokine VEGF (88).VEGF is a growth factor produced by macrophages (89), platelets (90), and keratinocytes (91). VEGF’s mode of action relies on increasing endothelial cell permeability through inducing the expression of cell adhesion molecules and recruiting monocyte and neutrophils to inflammatory sites (92). This dual role, in cardiovascular events and leukocyte chemoattraction, led researchers to further explore the association between VEGF and FMF. Interestingly, no studies assessed VEGF production in adult or pediatric FMF patients. However, several studies investigated the possibility of an association between *VEGF* genetic variations and FMF susceptibility. In a Turkish population, 105 patients with FMF and 100 controls were genotyped to assess whether the rs35569394 *VEGF* 18 bp I/D variant plays a role in FMF. The study including children and adult participants showed a significantly different distribution of genotypes between controls and patients. The *VEGF* D/D genotype was significantly higher in patients compared to healthy subjects, whereas the I/D genotype frequency was greater in controls. *VEGF* genotypes had also a different effect on the disease clinical manifestations where joint pain was found to be more common in patients with D/D genotype compared to the I/D genotype (93). These results suggest that the *VEGF* D/D genotype is associated with an increased risk and manifestation of FMF. In another study, the rs3025039 (+936C/T) functional polymorphism of *VEGF* gene was evaluated in order to determine if there is any association with susceptibility to FMF. Findings revealed that even though the TT genotype was present at higher frequencies in FMF patients than controls, the difference was not statistically significant. Additionally, there was no correlation between the tested polymorphism and FMF clinical manifestations, such as arthritis, abdominal pain, pleuritis, myalgia, arthralgia, and erysipelas-like erythema (94). As future directions, it might be interesting to study the effect of the genetic variations in *VEGF* on the protein levels in serum and supernatants of PBMCs and PMNs cultured *ex vivo* and subsequently to correlate levels with the phenotypic features of FMF.

### 3.8 Chemokines

The potential role of various chemokines, mainly CCL1, CXCL1, and CXCL16, in the pathogenesis of FMF has been also investigated. CXCL1 is produced by activated microglia and dependent on Th17 cells and IL-17 production, while CCL1 is expressed in monocytes, activated macrophages, and Th2 and Treg cells. Both chemokines induce a variety of activities including cytokine secretion and trafficking of immune cells (95, 96). CXCL16 is another member of the chemotactic cytokine superfamily. It is secreted by activated macrophages, fibroblasts, and dendritic cells and plays an important role in inflammatory processes as well as tissue damage and fibrosis (97). A Danish research team conducted a study to assess the diagnostic potential of a panel of 23 cytokines and chemokines associated with monocyte and macrophage function in FMF patients carrying variants of uncertain clinical significance (VUS) (53). Patients were grouped into three groups according to their genotypes and the majority were under colchicine therapy; group 1 included patients with VUS or no detected variants, and groups 2 and 3 comprised patients with monoallelic and biallelic *MEFV* pathogenic or likely pathogenic variants respectively (12). Monocytes enriched from PBMCs of patients and controls were stimulated to activate the pyrin inflammasome and the secretory profile was analyzed in cell supernatants. CCL1 and CXCL1 were the only different markers between the two groups and their levels were significantly decreased in FMF patients as compared to healthy subjects, which was unexpected considering that FMF attacks are characterized by high levels of infiltrating myeloid cells in synovial and serosal fluids, indicative of elevated chemokine-mediated trafficking. This result is contradictory to that noted by Koga et al. who reported elevated CXCL1 serum levels using multisuspension cytokine array in untreated FMF patients during attack and attack-free periods (59), suggesting that the reduced CCL1 and CXCL1 levels observed in the Danish study could be secondary to colchicine treatment. On the other hand, ROC analysis revealed that levels of CCL1 and CXCL1 could be used to distinguish FMF patients with VUS from healthy controls with 80% and 70% sensitivity, respectively (53). Therefore, the ability of CCL1 and CXCL1 to be used as discriminatory markers highlights their potential benefit in future functional diagnostic assays. In another research including 53 male FMF patients and 60 healthy controls, researchers evaluated CXCL16 levels by ELISA in the first 24 h of the attack (97). The levels of CXCL16 were significantly higher in FMF patients compared to controls; however, no correlation was detected between CXCL16 levels and attack frequency and disease duration. Moreover, ROC curve analysis revealed satisfactory score of sensitivity (83%) and specificity (68%) for CXCL16 and levels were predictive for monitoring inflammation in FMF patients. Accordingly, it was suggested that CXCL16 may be a promising novel diagnostic biomarker for FMF (97). Further prospective, randomized, large studies are warranted for elucidating the role of chemokines in the pathogenesis of FMF.

### 3.9 S100 protein family

The multifunctional role of S100 family of proteins has been well-established. These proteins are known to exhibit cytokine like properties and participate in a variety of biological activities including antimicrobial activities, calcium storage and transport, and most importantly inflammation activation and chemo-attraction (98). In fact, the involvement of S100 proteins in AIDs, especially FMF, has been attributed to potential dysregulations in the alternative secretory pathways of monocytes and granulocytes (99). Accordingly, several studies evaluated the expression of S100 proteins, predominantly S100A8, S100A9, and S100A12, and correlated levels with the severity of FMF in both adults and children. Kallinich et al. studied the serum levels of S100A12, erythrocyte sedimentation rate (ESR), C-reactive protein (CRP), and serum amyloid A (SAA) in 52 children and adults with FMF over 18 months (48). The age of the patients ranged between 3.2 and 20.4 years old with a median age of 10.3 years. Patients were divided into four groups: untreated FMF patients consisting of recently diagnosed patients who are not yet placed on colchicine, “stable FMF” treated with colchicine with well controlled disease, “unstable FMF” treated with colchicine and showing persistent symptoms, and *MEFV* mutation carriers. S100A12 proteins levels were found to be the highest in the untreated group with significant increase in levels as compared to all experimental groups and to controls. Furthermore, concentrations were higher in “unstable FMF” patients than in the “stable FMF” group in which levels were also above the normative age-matched pediatric cut-off value. Interestingly, unlike the classical markers of inflammation, CRP, ESR, and SAA, S100A12 was significantly elevated in clinically unaffected homozygous *MEFV* gene mutation carriers, supporting the ongoing subclinical inflammation in FMF. These results indicate that S100A12 is a highly sensitive diagnostic biomarker for monitoring disease activity, inflammation, and response to colchicine treatment in FMF. Stoler et al. further supported these findings by showing increased serum levels in patients than controls and a heightened *ex vivo* spontaneous release of S100A12 by FMF neutrophils that was later reduced by the addition of colchicine (62). In another study, researcher aimed to evaluate whether a potential relationship might exist between S100A12, Toll-like receptor 4 (TLR4) and the disease activity of both FMF and juvenile idiopathic arthritis (JIA) (100). The study employed 69 children with FMF, 68 children with JIA, and compared the serum levels of S100A12 and TLR4 (ELISA) to 35 healthy children. Levels of S100A12 were found to be significantly higher in children with FMF compared to controls. Although S100A12 levels dropped dramatically after administration of colchicine therapy, the researchers did not find any correlation between disease activity and S100A12 levels (100). Similar results were also detected in a cohort of 57 pediatric FMF patients (43 in attack-free periods and the remaining 14 in acute attack) and 31 healthy controls where S100A12 was significantly higher in acute FMF patients when compared to both attack-free FMF patients and controls. Additionally, levels were higher in attack-free period patients than in controls and in M694V homozygous patients as compared to those having other genotypes (51). S100A12 levels were also studied in other populations, namely, the Egyptian population. Abdallah et al. evaluated serum levels of S100A12 and Resolvin D1, a lipid mediator with anti-inflammatory activity, in 78 pediatric FMF patients, in the quiescent stage, and 60 age-and-sex-matched healthy controls using ELISA (101). The study aimed to assess the role of S100A12 and Resolvin D1 in the diagnosis and detection of subclinical inflammation in FMF children. Findings showed that S100A12 levels were significantly higher in colchicine treated children than controls. Furthermore, S100A12 was found to be a reliable biomarker of inflammation in pediatric FMF due to its high sensitivity (97.4%) and specificity (80%). Finally, a research team thought of assessing the effect of pyrin mutations on the secretion of S100A8/A9 alarmins *in vitro* and *ex vivo* (45). The study noted increased production of S100A8/A9 in serum and TcdA-induced PBMC of FMF patients compared to controls. Interestingly, S100A8/A9 complexes were found to directly interact with pyrin and their secretion was dependent on pyrin, caspase-1, and GSDMD (45).

Table 1 presents the main findings of the studies investigating cytokine expression and polymorphisms in FMF.

**Table 1:** Summary of studies assessing cytokine expression and polymorphisms in FMF patients as compared to controls and their role in disease susceptibility and/or severity.

Research studies	Participants	Objective(s)	Cytokine(s) studied	Experimental procedure(s)	Findings
Yigit et al. (54)	*N* = 62 FMF patients with amyloidosis *N* = 110 FMF patients without amyloidosis *N* = 120 healthy controls	Investigate whether genetic variations in *MIF*, *IL4*, and *IL-RA* genes influence the risk of developing FMF-related amyloidosis	MIF IL-4 IL-1RA	Genotyping of rs755622 *MIF* (−173G/C) by PCR-RFLP Genotyping of rs2234663 *IL-1RA* 86 bp VNTR and rs79071878 *IL-4* 70 bp VNTR by PCR	*IL-1RA* VNTR A1/A2, A1/A4 genotypes and A1 A4 alleles as well as the *MIF* (−137G/C) polymorphism exhibited higher frequency in both patient groups compared to controls *IL-4* VNTR P1 allele was more common in FMF patients with amyloidosis compared to controls; however, no significant difference was observed between groups of FMF patients
Jorch et al. (45)	N_1 _= 8 FMF patients (1 untreated, 7 treated with colchicine) N_1_= 25 healthy controls N_2_= 26 FMF patients N_2_= 29 healthy controls	Assess the effect of pyrin mutations on the secretion of S100A8/A9 alarmins	S100A8/A9	Stimulation of PBMC with TcdA (1*μ*g/ml) Quantification of S100A8/A9 in serum of supernatant of cells by ELISA	Increased production of S100A8/A9 in serum and TcdA-induced PBMC of FMF patients compared to controls S100A8/A9 complexes directly interact with pyrin and their secretion is dependent on pyrin, caspase-1, and gasdermin D
Dumur et al. (100)	*N* = 69 pediatric FMF patients *N* = 68 pediatric JIA patients *N* = 35 healthy controls	Investigate the possible relationship of serum S100A12 and TLR4 with FMF activity	S100A12	ELISA	Serum S100A12 levels were significantly higher in FMF patient group than in both the JIA and control groups Non-significant increase of S100A12 levels in the attack period compared to the attack-free period S100A12 levels dropped dramatically after administration of colchicine therapy but no correlation was detected between S100A12 levels and FMF activity
Sezer et al. (93)	*N* = 105 FMF patients *N* = 100 healthy controls	Assess whether the rs35569394 *VEGF* 18 bp I/D variant play a role in FMF	VEGF	PCR	VEGF D/D genotype was significantly higher in patients compared to controls, whereas the I/D genotype frequency was greater in controls Joint pain was found to be more common in patients with D/D genotype compared to the I/D genotype
Martirosyan et al. (56)	*N* = 35 FMF patients (21 free of attacks and 14 in acute attack) *N* = 20 healthy controls	Assess whether cytoskeletal dynamics in the absence of pathogens may cause the abnormal activation of PMNs in FMF	IL-1β	Stimulation of neutrophils with fMLP (100 ng/ml) Quantification of IL-1β by ELISA	Spontaneous and induced activation of patients’ neutrophils after transmigration and increased production of IL-1β
Cakan et al. (85)	*N* = 98 pediatric FMF patients *N* = 157 healthy controls	Assess the potential association between rs755622 (−173G/C) polymorphism in *MIF* gene and disease susceptibility of children with FMF	MIF	PCR-RFLP	Individuals with the CC allele seem to have a higher predisposition to FMF No significant difference between patients and controls in terms of allele frequency
Akyol et al. (97)	*N* = 53 male FMF patients *N* = 60 healthy controls	Investigate the relationship between CXCL16 levels and FMF disease Evaluate CXCL16 levels as a novel biomarker for FMF	CXCL16	ELISA Assessment of sensitivity and specificity by a ROC curve analysis	CXCL16 levels were significantly higher in serum of FMF patients than controls No correlation between CXCL16 levels and attack frequency and disease duration The cut off value of CXCL16 was 2.68 ng/ml with 83% sensitivity and 68% specificity Logistic regression analysis indicated that high CXCL16 was a predictive parameter for FMF disease
Abdallah et al. (101)	*N* = 78 attack-free pediatric FMF patients *N* = 60 healthy control	Study the role of S100A12 and resolvin D1-related genes and serum levels in the diagnosis and detection of subclinical inflammation in FMF children during attack-free period	S100A12	Quantification of S100A12 by ELISA Assessment of sensitivity and specificity by a ROC curve analysis	S100A12 was significantly increased in colchicine treated children than controls. S100A12 is a reliable biomarker of inflammation in pediatric FMF with high sensitivity (97.4%) and specificity (80%)
Türkmenoğlu et al. (51)	*N* = 57 pediatric FMF patients (43 in an attack-free period and 14 in an attack period) *N* = 31 healthy children	Investigate the relationship of S100A12 with attacks and inflammatory events in FMF patients	S100A12	ELISA	S100A12 levels were significantly higher in serum of acute FMF patients when compared to both attack-free FMF patients and controls Levels were also higher in attack-free period patients than in controls and in M694V homozygous patients as compared to those having other genotypes
Koga et al. (65)	*N* = 28 FMF patients *N* = 22 patients with sepsis *N* = 118 healthy controls	Identify potential biomarkers to distinguish FMF from sepsis	GM-CSF, TNF-α, FGF-2, G-CSF, GRO, IFN-γ, IL-17A, IL-6, IL-8, IP-10, MCP-1, MIP-1α, MIP-1β, VEGF, and IL-18	Ranking cytokines by importance using a multivariate classification algorithm Performing a logistic regression analysis to determine specific biomarkers for discriminating FMF from sepsis Identification of specific molecular networks by cluster analysis of each cytokine	GM-CSF, FGF2, VEGF, MIP-1β, and IL-17 were elevated in FMF compared to sepsis TNF-α was increased in sepsis compared to FMF Measurement of both GM-CSF and TNF-α could distinguish FMF from sepsis with high accuracy (cut-off values for GM-CSF = 8.3 pg/ml; TNF-α = 16.3 pg/ml; sensitivity, 92.9%; specificity, 94.4%; accuracy, 93.4%)
Mortensen et al. (53)	*N* = 23 FMF patients *N* = 6 healthy controls	Identify pyrin inflammasome specific mechanisms to improve FMF treatment and diagnosis	IL-1β IL-1Rα IL-1RN	Stimulation of monocytes with C3 toxin (1 μg/ml) and LPS (10 ng/ml) Quantification of cytokines by RT-PCR and Luminex magnetic assay or ELISA	Reduction of *IL-1RN* mRNA expression and IL-1Rα secretion in healthy controls and FMF monocytes by pyrin activation Decreased ratio of IL1Rα/IL-1β in colchicine-treated patients and decreased secretion of IL1-Rα compared to healthy controls
Martirosyan et al. (56)	*N* = 35 FMF patients (21 in attack-free and 14 in acute flares) *N* = 20 healthy controls	Assess whether cytoskeletal dynamics in the absence of pathogens may cause the abnormal activation of PMNs in FMF	IL-1β	Stimulation of neutrophils with fMLP (100 ng/ml) Quantification of IL-1β by ELISA	Spontaneous and induced activation of patients’ neutrophils after transmigration and increased production of IL-1β
Yıldırımtepe Çaldıran et al. (57)	*N* = 60 FMF patients *N* = 30 healthy controls	Investigate the relationship of IL-1β, IL-18, and caspase-1 with disease severity and acute phase reactants in FMF	IL-18 IL-1β	ELISA	IL-1β and IL-18 levels were higher in serum of FMF attack patients compared to controls IL-1β production was positively correlated with acute phase reactant levels and disease severity scores in patients
Wada et al. (61)	*N* = 12 FMF patients with *MEFV* exon 10 mutations	Evaluate the longitudinal expression of IL-18 upon treatment with colchicine	IL-18	ELISA	Serum levels of IL-18 were elevated in FMF attack and attack-free patients FMF patients without *MEFV* mutations exhibited atypical FMF phenotype with lower IL-18 elevation Serum IL-18 declined progressively after colchicine treatment in seven patients IL-18 levels showed continuous elevation in five patients despite declines in IL-6
Stoler et al. (62)	*N* = 12 FMF patients *N* = 6 patients with diverse inflammatory diseases *N* = 9 healthy controls	Characterize neutrophilic inflammatory responses in FMF Delineate gene–dose effects on a cellular level	IL-18 S100A12	Stimulation of neutrophils with PMA (10 nM) or LPS (10 ng/ml) Quantification of cytokines by RT-qPCR and ELISA	Significant increase of IL-18 and S100A12 in serum and supernatant of neutrophils of patients with homozygous M694V mutations compared to controls and other patients Gene-dose effect exhibited by an increased IL-18 secretion in patients with two mutations compared to those with a single mutation No reduction in the secretory potential of neutrophil after blocking IL-1β, indicating that neutrophil activation is independent from IL-1 activation
Ibrahim et al. (58)	*N* = 25 FMF patients *N* = 25 healthy controls	Evaluate *RAC1* expression and role in IL-1β and caspase-1 production as well as oxidative stress generation in FMF	IL-1β IL-6	Stimulation of PBMCs and PMNs with LPS (1 µg/ml) Quantification of cytokines by ELISA	FMF patients during attacks had significantly higher *RAC1* expression levels than patients between attacks or controls No difference in *RAC1* expression levels between attack-free patients and healthy subjects Significantly higher IL-1β levels were detected in LPS-stimulated PBMCs of FMF patients during attacks compared to attack-free patients or controls IL-6 levels were comparable in unstimulated PBMC culture supernatants of attack-free patients and controls RAC1 inhibition reduced IL-1β levels but not IL-6
Jamilloux et al. (63)	*N* = 48 FMF patients *N* = 26 healthy controls	Investigate whether *MEFV* mutations impact pyrin's ability to detect RhoGTPase inhibition and other inflammasome stimuli	IL-18 IL-1β	Stimulation of monocytes isolated from PBMCs by TcdB (125 ng/ml) Quantification of IL-18 and IL-1β by ELISA	Stimulated monocytes secreted more IL-18 and IL-1β than controls Monocytes from patients carrying two *MEFV* mutations displayed an increased pyrin inflammasome response compared with monocytes from patients with a single mutation *MEFV* mutations decreased the activation threshold of pyrin inflammasome which might explain the increased release of IL-18 and IL-1β in FMF patients
Marzouk et al. (49)	*N* = 50 pediatric FMF children *N* = 40 healthy controls	Investigate the relationship between IL-4 levels, *IL4* VNTR, and FMF occurrence, severity, and response to treatment in Egyptian FMF children	IL-4	Quantification of IL-4 levels by ELISA Genotyping of *IL-4* VNTR by PCR	No significant difference in genotype distribution of *IL-4* VNTR between patients and controls No significant difference in serum IL-4 levels in children with FMF attack and those in attack-free period compared to controls No correlation between *IL-4 VNTR* and FMF severity or response to colchicine therapy
Koga et al. (59)	*N* = 75 FMF patients *N* = 40 healthy controls	Identify specific biomarkers to diagnose or assess disease activity in FMF patients	IL-1β, IL-1RA, IL-2, IL-4, IL-5, IL-6, IL-7, IL-8, IL-10, IL-12 (p40), IL12 (p70), IL-17, IL-18, TNF-α, IFN-α, IFN- γ, GM-CSF, G-CSF, VEGF, sCD54, sCD106, FGF2, CCL2, CCL3, CCL4, CCL22, CXCL1, CXCL10, and CX3CL1	Quantification of serum levels by multisuspension cytokine array Ranking cytokines by importance using a multivariate classification algorithm Performing a logistic regression analysis to determine specific biomarkers for discriminating FMF patients in attack Identification of specific molecular networks by cluster analysis of each cytokine	The combined measurement of IL-6, G-CSF, IL-10, and IL-12p40 discriminated febrile attack periods from attack-free periods The combined measurement of IL-6, IL-18, and IL-17 distinguished FMF patients in attack from the controls
Özer et al. (50)	*N* = 99 pediatric FMF patients *N* = 157 healthy controls	Identify relationship between rs1800795 *IL-6* (−174G/C) polymorphism and clinical features, disease severity score, and proteinuria in FMF children	IL-6	PCR-RFLP	*IL-6* (−174 G/C) genotype and allele frequencies were comparable between patients and controls No correlation was detected between (−174G/C) polymorphism and frequency of FMF clinical features or disease severity score
Nursal et al. (55)	*N* = 160 FMF patients *N* = 120 healthy controls	Investigate the relationship between *IL-1RA* and *IL-4* and risk of FMF	IL-1RA IL-4	Genotyping of *IL-1RA* VNTR and *IL-4* VNTR by PCR	P1P1 genotype frequency was higher in patients than controls No significant difference in allelic frequency of the *IL-4* VNTR polymorphism between controls and patients *IL-1RA* VNTR was not associated with FMF risk
Ibrahim et al. (26)	*N* = 42 FMF patients *N* = 42 controls	Determine a potential association of *IL-1β* and *IL-1RA* gene polymorphisms with occurrence and/or severity of the disease	IL-1ß IL-1RA	Stimulation of monocytes by LPS (1 μg/ml) Quantification of IL-1β and IL-1RA levels in supernatants of PBMC cultures by Luminex Multiplex technology Genotyping of *IL-1β* (−511C/T), *IL-1β (−31 T/C)*, and *IL-1β* (+3954 T/C) by PCR-RFLP and *IL-1RA* VNTR by PCR	CC genotype and C allele at positions −31 and +3,954 were significantly higher in FMF patients than controls Patients with *IL-1β* −31CC genotype have an increased LPS-induced IL-1β secretion and a higher disease severity score when compared to patients carrying TC and TT genotypes
Ibrahim et al. (20)	*N* = 34 FMF patients (9 during attacks and 25 attack-free) *N* = 24 healthy controls	Evaluate the *ex vivo* cytokine profile of FMF patients during acute attacks and attack-free periods to clarify the inflammatory mechanism underlying FMF	IL-1β, IL-1α, IL-1RA, IFN-γ, IL-4, IL-17, IL-22, IL-6, and TNF-α	Stimulation of monocytes by LPS (1 μg/ml) and T lymphocytes by anti-CD3/CD28 beads (2.5 × 10^4^ beads/10^6^ cells/ml) Quantification of cytokines in serum and supernatants of PBMCs by Luminex Multiplex technology	Only IL-6 and TNF-α serum levels were significantly higher in FMF patients than controls LPS-stimulated PBMCs of FMF attack patients produced higher amounts of IL-6, TNF-α, IL-1β and IL-1α as compared to attack-free FMF patients and to healthy subjects Unstimulated and anti-CD3/CD28-stimulated PBMC supernatants had the lowest levels of IFN-γ and IL-4 as compared to attack-free patients and to controls Anti-CD3/CD28-stimulated PBMC supernatants of FMF patients during and between attacks had greater levels of Th17 cytokines IL-17 and IL-22 IL-1β, IL-1α, IL-6, IL-17, and IL-22 production was increased in M694V homozygous FMF patients when compared to those heterozygous for the same mutation or with other genotypes
Savran et al. (46)	*N* = 51 FMF patients *N* = 30 healthy controls	Evaluate the serum levels of MIF protein in FMF patients	MIF	ELISA	MIF levels were significantly higher in patients than controls
Bonyadi et al. (74)	*N* = 86 M694V homozygous FMF patients *N* = 100 healthy controls	Evaluate the role of rs1799964 *TNF-*α (−1,031 T/C) and rs1800629 *TNF-*α (−308G/A) polymorphisms in patients with FMF	TNF-α	PCR-RFLP	Both polymorphisms showed no significant difference in genotype and allele distribution between patients and controls Patients with TNF-α −308 GG are more susceptible to develop amyloidosis and arthritis whereases TNF-α −308 A may have a protective role among M694V homozygous patients with amyloidosis *TNF-*α (−1,031 T/C) did not reveal a significant correlation with the manifestation and progression of clinical characteristics of FMF
Kallinich et al. (48)	*N* = 52 pediatric FMF patients treated with colchicine	Analyze the role of S100A12 in the detection of inflammation in patients with FMF	S100A12	ELISA	Serum S100A12 levels were the highest in untreated FMF patients S100A12 decreased significantly after introduction of colchicine therapy Serum concentrations of S100A12 was significantly higher in patients with persistent symptoms than in those with clinically controlled disease
Gunesacar et al. (94)	*N* = 75 FMF patients *N* = 122 healthy controls	Evaluate rs3025039 *VEGF* (+936C/T) polymorphism in susceptibility to FMF	VEGF	PCR-RFLP	TT genotype was present at higher frequencies in FMF patients than controls but not statistically different No correlation was found between *VEGF* (+936C/T) and FMF clinical manifestations such as arthritis, abdominal pain, pleuritis, etc.
Celebi Kobak et al. (73)	*N* = 41 FMF patients *N* = 43 healthy controls	Evaluate rs361525 *TNF-*α (−238G/A) and rs1800629 *TNF-*α (−308G/A) genotype and allele distribution in patients with FMF	TNF-α	Amplification refractory mutation system polymerase chain reaction	No significant difference in genotype and allele frequencies of both polymorphisms between patients and controls No association between genotypes of both polymorphisms and the frequency of acute attacks in FMF
Karahan et al. (66)	*N* = 156 FMF patients (80 with amyloidosis) *N* = 90 healthy controls	Investigate the role of rs1800795 *IL-6* (−174 G/C) polymorphism in the clinical outcome of FMF and amyloidosis	IL-6	PCR-RFLP	*IL-6* (−174 G/C) polymorphism is not associated with FMF nor amyloidosis with genotypic and allelic frequency distribution being similar in patients, with and without amyloidosis, and controls
Akar et al. (70)	*N* = 126 FMF patients (45 with amyloidosis) *N* = 79 healthy controls	Explore the association of rs1800629 *TNF-*α (−308G/A) with FMF and associated amyloidosis	TNF-α	PCR-RFLP	No significant difference in the distribution of the *TNF-*α *−*308A allele between patients and controls or between patients with and without amyloidosis

CCL, CC chemokine ligand; CXCL, CXC motif chemokine ligand; ELISA, enzyme-linked immunosorbent assay; FGF, fibroblast growth factor; FMF, familial Mediterranean fever; fMLP, N-Formylmethionine-leucyl-phenylalanine; G-CSF, granulocyte colony-stimulating factor; GM-CSF, granulocyte-macrophage colony-stimulating factor; GRO, growth-regulated protein alpha precursor; IFN, interferon; IL, interleukin; IP, interferon γ-inducible protein; JIA, juvenile idiopathic arthritis; LPS, lipopolysaccharide; MCP, monocyte chemoattractant protein; MIF, macrophage migration inhibitory factor; MIP, macrophage inflammatory protein; PCR, polymerase chain reaction; PBMC, peripheral blood mononuclear cells; PMN, polymorphonuclear neutrophils; PMA, phorbol myristate acetate; RAC, ras-related C3 botulinum toxin substrate 1; RFLP, restriction fragment length polymorphism; ROC, receiver operating characteristics; RT-qPCR, reverse transcription-quantitative polymerase chain reaction; sCD, soluble cluster of differentiation; Th, T helper cells; Tcd, Clostridium difficile toxin A; TNF, tumor necrosis factor; TLR, toll-like receptor; VEGF, vascular endothelial growth factor; VNTR, variable number of tandem repeats.

## 4 Anticytokine therapeutics in FMF

FMF treatments aim to improve patient's quality of life by reducing inflammation, the frequency of attacks, and the risk of secondary amyloidosis (102). Since 1972, colchicine is considered the mainstream therapeutic option for FMF patients (27). In fact, colchicine is FDA approved for patients older than 4 years (103). Moreover, its safety and efficacy have been established in pediatric FMF patients below 4 years (103). Nevertheless, approximately 5%–10% of FMF patients are considered as resistant to colchicine while 2%–5% are known to be intolerant due to colchicine adverse effects (30); this percentage might reach approximately 20% according to a large cohort study conducted in Anatolia (31). Therefore, intervention with biologics targeting cytokines seems to be a reasonable alternative for FMF patients that are refractory to colchicine.

In this part of the review, we will be thoroughly discussing the main studies tackling biologics as treatment in FMF children, namely, anti-IL-1 (anakinra, canakinumab, and rilonacept), anti-TNF-*α* (etanercept, adalimumab, and infliximab), and anti-IL-6 notably tocilizumab (Figure 1). Anti-IL-1 drugs are the primary biological therapy used in pediatric FMF patients (36, 104) with their first administration being reported in the literature in 2007 (105, 106). Since then, more than 30 studies have been conducted, mainly in Turkey, to establish the efficacy, safety, and side effects of anticytokine therapeutics and their impact on the life of pediatric FMF patients. Due to the significant amount of research on this topic, this paper will only focus on the most recent and relevant studies conducted on children in the last 10–15 years.

### 4.1 Anti-IL-1 therapeutics

The main IL-1 inhibitors used in FMF patients are canakinumab and anakinra. Canakinumab is a human monoclonal antibody specifically targeting IL-1β and blocking its interaction with IL-1R and the downstream inflammatory signaling (107). It is the only FDA and EMA approved cytokine blocker for treatment of FMF and can be administered subcutaneously every 4–8 weeks (108). On the contrary, anakinra, a human recombinant unglycosylated analog of IL-1RA, is only approved for clinical use by EMA and administered daily by subcutaneous injection (107, 108). Beside canakinumab and anakinra, IL-1 activity is inhibited by rilonacept whose usage for FMF patients is still not yet approved. Rilonacept is a dimeric fusion protein made of the extracellular domain of IL-1R1 and the constant domain of human immunoglobulin G1, targeting IL-1β and preventing its binding to IL-1R1 (107).

Aydin et al. characterized 33 FMF children receiving an anti-IL-1 treatment by comparing their genetic and clinical profiles to a total of 542 recruited pediatric FMF patients (109). Treated patients had earlier disease onset along with severe and frequent clinical manifestations such as acute arthritis, chest pain, and erysipelas-like erythema. In fact, 17.2% of patients under IL-1 inhibitors were already suffering from amyloidosis with 82.8% of them being resistant to colchicine. Furthermore, almost every child treated with anti-IL-1 in this study had at least a single M694V mutation. Such findings may be supported by the genotype-phenotype correlation research conducted on Turkish children with FMF (110). The retrospective study of 102 FMF patients, divided into three groups based on M694V mutation, showed that homozygosity to M694V mutation increases the risk to early disease onset, severe disease manifestations, resistance to colchicine, and secondary amyloidosis. Therefore, it can be concluded that FMF patients with the above-mentioned characteristics may show a higher dependency on IL-1 inhibitors (109). In fact, anti-IL-1 treatment is even more tailored and designed individually for each patient compared to colchicine treatment that is dependent on the patient’s response and tolerance (111). For instance, in the study of Eroglu et al., involving 14 children and adolescents, 11 patients were under anakinra treatment for an average period of 8 months and three were initially administered etanercept but then switched directly to canakinumab (111). At the third month, nine patients responded well to anakinra while the others switched to canakinumab due to noncompliance, local side effects, and active arthritis. However, only five patients decided to continue anakinra treatment and consequently canakinumab was used in nine patients, all of whom showed a positive response to the treatment. During follow-up, canakinumab dose was increased in two patients whereas the dosing interval was extended to every 12–16 weeks in three patients. In addition, three patients stopped receiving anti-IL-1 treatment as they showed a good response with colchicine only. The remaining patient initially under steroid treatment and experiencing frequent protracted febrile attacks was administered two doses of canakinumab with 8 months interval. After each dose, the patient showed positive response within 2 days and did not require further steroid therapy. Thus, this study highlights the differences in drug administration and duration between FMF patients indicating the need for personalized treatment protocols.

The safety and efficacy of anti-IL-1 therapeutic agents have been well-established in the general FMF population (104, 112). Recently, studies conducted on pediatric FMF patients showed similar safety and efficacy profile of anakinra and canakinumab. Erkilet et al. evaluated the efficacy and safety of IL-1 inhibitors in colchicine resistant FMF children (102). Among 656 pediatric patients retrospectively studied, 27 patients were under anakinra or canakinumab treatment. From the 27 patients, 66% were homozygous for M694V mutation with 100% of the patients showing a significant decrease in the frequency and duration of attacks. Interestingly, in patients using anakinra, the number of attacks was reduced by 83.2% sixth month after treatment and by 88% after one year. However, the number of attacks per year was less in patients treated with canakinumab with a significant decrease in the frequency of attacks by 93.9% and 94.6% sixth months and 12 months post-treatment respectively. In terms of safety, only a single bronchopneumonia incident and three local injection site reactions were reported suggesting an overall safe profile of anakinra and canakinumab in this cohort of pediatric FMF patients (102). Accordingly, the researchers concluded that canakinumab have a similar safety but a higher efficacy in reducing FMF attacks as compared to anakinra (102). These results are not aligned with data reported by Ayaz et al. on 26 FMF children resistant to colchicine as both anakinra and canakinumab were good and similarly efficient in reducing disease severity (113).

In 2020, Sag and colleagues evaluated the efficacy and safety of anti-IL-1 treatments in 40 pediatric FMF patients, of whom 38 were homozygous for the M694V mutation (114). IL-1 inhibitors were administered continuously in 34 patients and on demand in 6 patients; 28 patients were on canakinumab while the remaining 6 on anakinra. Before treatment, the frequency of attacks was around one episode per month along with high CRP levels. However, 6 months post-treatment, there was a decrease in the number of attacks and CRP levels which persisted for an average of four years during the follow-up period. In addition, the severity of attacks was reduced. Most of the patients suffered from an abdominal pain before anakinra or canakinumab usage which was resolved upon anti-IL-1 administration. Regarding the safety assessment, the researchers reported that during the course of treatment, there was three hospitalization events caused by mild infections, 11 cases of local skin reactions along with two leucopenia reports in patients using anakinra, and one thrombocytopenia incidence in a patient treated with canakinumab (114). Interestingly, this study reported a first time on-demand use of anti-IL-1 treatment in adolescent females that were only under severe attacks during the menstrual period. Such administration has been significantly beneficial for these patients with no increase in CRP levels being reported. Therefore, this study provided insights into how adolescent females experiencing attacks exclusively during their menstrual cycle may benefit from on-demand therapy (114).

Anti-IL-1 agents were evaluated as well in Basaran et al. study in which eight pediatric FMF patients were considered refractory to colchicine because of the continuous severe attacks even after having the maximum colchicine dose (2 mg/day) (115). Similar to the genetic profile of other colchicine resistant patients, six out of eight patients were M694V homozygotes. In all eight children, anakinra has been used with a subcutaneous dose starting from 1 mg/kg/day and increased if needed to 3 mg/kg/day. After 6 months of administration, four of the six patients switched to canakinumab treatment refusing daily injection. Overall, Basaran et al. findings revealed that both drugs were beneficial with no severe adverse effects (115). Discontinuing colchicine treatment in three patients three months after canakinumab use resulted in an increase in CRP levels indicating that canakinumab alone was not enough to control inflammation (115). This finding is in line with that of Yildirim et al. who recently reported a lack of knowledge about the long-term efficacy of anti-IL-1 drugs and suggesting the administration of a maximal tolerated dose of colchicine to FMF patients treated with IL-1 antagonists (108). It is noteworthy to mention that switching from anakinra to canakinumab is frequently reported in many studies and it is mainly due to inadequate reaction, side effects such as reactions at the injection site, ineffectiveness, or lack of daily access (105). Therefore, as an alternative, Cebeci et al. aimed to assess the efficacy of a single dose administration of anakinra in reducing the severity, duration of attacks, and hospitalization in FMF patients with a median age of 13 years old (116). In fact, typical FMF attacks lasted more than 24 h, however, with the single anakinra dose, most of the episodes ended in a shorter duration ranging from 10 min to 4 h.

In 2016, a study aimed to assess the response of two pediatric FMF patients groups treated with anti-IL-1 drugs (117). The first group included a total of seven children refractory to colchicine while the second comprised six children with FMF related amyloidosis. In group 1, five out of seven patients were homozygous for the M694V mutation and almost all patients had around 1–3 episodes in a month. The administration of anakinra and canakinumab along with colchicine resulted in a decrease in attack frequency from an average of 36 attacks per year to an average of 0–4 attacks post-treatment. Moreover, the average CRP levels decreased from 90 mg/L to 2.7 mg/L after treatment. In this group, the safety assessment of anti-IL-1 drugs showed no side effects in all the seven children upon 9–23 months of follow-up. In group 2, the six children with amyloidosis were using anakinra with one patient suffering from a nephrotic syndrome, two with chronic kidney disease, and three with renal transplantation. It is important to note that anakinra was preferred over canakinumab in this group of patients because of the high cost of canakinumab (117). The daily injections of anakinra were well tolerated in all the patients enrolled in the study. Treatment resulted in an increase in total protein from 3.4 g/dl to 5.8 g/dl and albumin from 0.8 g/dl to 3.4 g/dl. In addition, the 24 h urine protein excretion was reduced from 190 mg/m^2^/h to 12 mg/m^2^/h indicating an improvement in renal function. Moreover, no FMF attacks and amyloidosis-related gastrointestinal system findings were reported in the two patients with chronic kidney disease and a partial recovery was exhibited in the patient with nephrotic syndrome after anakinra therapy; thus, children were able to perform their daily tasks normally. Although amyloidosis is more commonly reported in adults as compared to children (118), it remains the most serious life-threatening complication of FMF. In this respect, this study sheds light on the possible occurrence of amyloidosis in FMF children and the importance of studying these rare cases, especially in patients that are non-responsive to colchicine.

The efficacy of canakinumab in managing FMF complications was also a topic of interest for Kışla Ekinci and her colleagues. Canakinumab was administered to 14 pediatric FMF patients who were selected for treatment because of colchicine resistance (11 patients) or for suffering from renal amyloidosis (one patient) or arthritis (two patients) (119). Canakinumab was completely efficient in 71.5% of patients with a significant reduction in the number of attacks, proteinuria, and levels of CRP without any reported adverse effects. These findings are in agreement with those reported in a research study conducted by Dr. Sami Ulus Children's Hospital in Turkey between January 2012 and January 2017. The study included 10 FMF children homozygous for the M694V mutation, of whom seven were refractory to colchicine and three with chronic kidney disease, amyloidosis, and nephrotic syndrome (120). Upon administration of canakinumab for an average duration of 21 months, all the patients were completely responsive with an overall decrease in inflammation and an improvement in renal function in FMF-associated amyloidosis patients. Regarding the safety of canakinumab in this group of patients, no direct adverse events were mentioned. Only a single pneumonia incidence upon an upper respiratory tract infection was reported without a need to discontinue the treatment. This study further supports reported data on the favorable effect of canakinumab in the management of FMF in pediatric patients that are either resistant to colchicine (121) or suffering from amyloidosis (117). Besides the Turkish population, canakinumab safety and efficacy was also confirmed in other populations. For instance, a 6-month open-label study was conducted on 7 Caucasian FMF children administered canakinumab in combination with colchicine (122). Patients received three subcutaneous injections (distant 4 weeks) of 2 mg/kg dose which was doubled if an attack occurred. In six out of seven patients, the rate of FMF attacks decreased by more than 50% and in three patients no attacks were reported.

Negative growth is another complication that may affect FMF pediatric population. Children's growth and development problems have been associated with multiple chronic diseases (123, 124) and mainly attributed to the high levels of cytokines affecting the growth hormone and insulin-like growth factor 1 (125). Interestingly, it was shown that colchicine administration may restore growth in FMF patients through management of attacks and inflammation (126, 127). Particularly, in the study conducted by Zung et al., the researchers demonstrated that starting colchicine treatment early on leads to a more significant impact on children’s growth. This is because the detrimental effects of inflammatory diseases on the linear growth of patients are resolved at an early stage (128). However, in patients resistant or intolerant to colchicine, no significant growth improvement was shown, hence anti-IL-1 therapy was recommended to control the patients’ inflammation (126). Indeed, a recently published study by Kadir et al. evaluated the long-term body weight and height changes in 22 FMF children treated with anakinra and/or canakinumab (129). Such treatment resulted in an overall complete attack-free in 80% of patients with no major side effects. Interestingly, upon treatment, the researchers reported a significant increase in height percentile from 19.6% ± 16% to 30.8% ± 23% and in weight percentile from 29.5% ± 30% to 39.1% ± 36%. Similar findings were also reported by Balci et al. who observed a positive change in both the height and body weight Z-scores in 11 FMF children treated with canakinumab and followed up for 1.5 years (130). On the contrary, Yucel et al. reported only an improvement in body weight Z-score with no increase in the linear growth of FMF patients under canakinumab treatment (121). In fact, the main reason behind the non-significant variation in the height percentiles may be the delay in starting canakinumab treatment or insufficient follow-up period. Therefore, it can be concluded that early intervention through anti-IL-1 agents and close follow-up are required in order to significantly enhance children's growth via efficient early management of inflammatory attacks.

Despite the well-established safety and effectiveness of canakinumab in FMF (131), evaluation of the long-term effect of canakinumab in pediatric FMF patients is limited to a few studies. In fact, a recent longitudinal study assessed the efficiency of canakinumab administration over an period of 23.9 months in 15 Turkish FMF patients (132). Fourteen out of the 15 FMF patients showed complete attack-free at 12 months treatment. The levels of SAA, CRP, and ESR were significantly reduced after canakinumab use. As for the safety follow-up, no severe side effects were reported with the exception of one hospitalization incidence due to bronchopneumonia and two incidences of teeth abscess. The findings of this study are in line with those reported in Laskari et al. longitudinal retrospective study on 14 adult and adolescent FMF patients (133). Upon receiving canakinumab for an average of 18 months, 11 patients were completely recovered within two months while the remaining three patients achieved partial recovery. The researchers reported that inflammatory markers were normalized to 92% at three months. Also, in the four patients that re-experienced attacks, canakinumab administration interval was shortened whereas in two patients with complete attack-free, the interval was safely increased. Therefore, we can conclude that the long-term efficacy and safety of canakinumab use in children with FMF requires further attention and future studies should focus on a larger number and more heterogenous group of pediatric patients (132).

Researchers were also interested in defining the most adequate dose, treatment duration, and interval time between canakinumab administration (134). In this regard, Akarcan and her colleagues evaluated the success of a standardized treatment protocol applied at their pediatric rheumatology department in nine FMF children with colchicine resistance (134). Initially, all patients received a monthly dose of canakinumab over the first 6 months. This was followed by bimonthly administration of canakinumab for the next 6 months. After a total of nine doses, its administration was ceased, and the patients were monitored for attacks. Those who experienced new attacks received canakinumab again at 3-month intervals. Such protocol seemed promising as upon stopping canakinumab treatment, few numbers of attacks were reported and resolved by re-administration of canakinumab at 3-month intervals again. Sener et al. proposed a similar protocol for canakinumab treatment management (135). Fifty-eight pediatric FMF patients were first administered a monthly dose of canakinumab over 6 months, followed by a bimonthly dose for the next 6 months and a final dose every 3 months over the last 6-month period. The results of this study showed that canakinumab administration was efficiently stopped in more than 64% of the patients in 18 months. These findings suggest that the feasibility of canakinumab withdrawal in colchicine resistant FMF patients is not well-defined and requires further investigation.

Finally, since FMF patients, especially children, are characterized by poor quality of life due the frequent recurrent episodes, a research conducted by Kurt et al. aimed to assess the quality of life and school attendance in FMF children under anti-IL-1 treatment (136). In this study, the treatment of 25 pediatric colchicine refractory FMF patients with anakinra or canakinumab resulted in a decrease in FMF attacks and AIDAI (auto-inflammatory diseases activity index) score which indicates an increase in quality of life along with a significant increase in school attendance. Interestingly, in Köhler et al. study, patients receiving canakinumab had a higher quality of life than those receiving anakinra because of daily injections (137).

Another anti-IL-1 therapeutic agent of interest that is worth discussing in this review is rilonacept. As compared to anakinra and canakinumab, rilonacept safety and efficacy in FMF is not well-elucidated; moreover, its administration in children is limited to a single study conducted in 2011. In fact, in their research, Hashkes et al. performed a randomized trial in which 12 FMF colchicine refractory patients aged 4 years or older with at least a single attack per month were administered rilonacept (138). Overall, the use of rilonacept decreased the number of FMF attacks; however, such findings were not representative, and more studies are needed to better elucidate the efficacy and safety of rilonacept in FMF patients, particularly among the pediatric FMF population.

### 4.2 Anti-TNF-α and anti-IL-6 therapeutics

Other biological drugs, namely, anti-TNF-*α* molecules (etanercept, adalimumab, and infliximab) and anti-IL-6 such as tocilizumab, have been used to treat FMF but not as extensively as IL-1 inhibitors. In general, anti-TNF-α molecules administration in FMF children failed to show promising results. For instance, the study of Ozen et al. showed that etanercept administration to five colchicine resistant FMF children was ineffective (139). The attacks frequency was reduced from 3 to 4 attacks per month to 2 attacks per month, however, they were not resolved completely. Therefore, the patients switched to anakinra instead. In accordance with these findings, a study conducted by Eroglu and colleagues revealed that out of the 14 enrolled pediatric FMF patients, three had to switch to anti-IL-1 therapy due to inadequate response and side effects (111). Ozgur et al. also reported similar observations in 35 patients (children and adults) who were originally administered etanercept, infliximab, or adalimumab and then asked to switch to anti-IL-1 therapy (36). Nevertheless, infliximab was found to be efficient in treating amyloidosis and protracted arthritis in a 12-year-old girl diagnosed with FMF (140). Moreover, Özçakar et al. reported alleviation of gastrointestinal symptoms, protracted arthritis, and nephrotic syndrome in four FMF pediatric patients with amyloidosis treated with infliximab for an average of 2–6.5 years (141). Additionally, by managing amyloidosis, infliximab administration led to an improvement in life quality, a decrease in hospitalization incidences, and a resumption of normal activities such as school or work.

Finally, to the best of our knowledge, there are no studies investigating the efficacy and safety of the anti-IL-6 agent tocilizumab in FMF children. Available reports suggest a promising effect of tocilizumab in the management of amyloidosis in FMF. For example, a very recent study conducted by Henes et al. reported a decrease in SAA in 25 adult FMF patients (37). In this regard, further studies are recommended in order to precisely evaluate the efficacy and safety of anti-TNF-α and anti-IL-6 drugs in the management of FMF severity and complications, mainly amyloidosis, among children.

## 5 Conclusion

New insights into cytokine profile and signature in FMF patients provide an intriguing perspective for finding potential pathways and mechanisms involved in FMF pathogenesis. Furthermore, designing studies that target specifically children would ensure that the way is paved for earlier and more accurate diagnostic and therapeutic strategies that could improve patients’ quality of life. That being said, it is imperative to highlight that studying cytokines in pediatric FMF is challenging and presents some limitations. First, studies assessing cytokine expression in pediatric FMF are limited in number. Actually, collecting samples from children may raise ethical issues and it requires parents’ approval which makes children enrollment difficult. Second, research in this area covers only a limited range of cytokines or explores one to two genetic polymorphisms which make the analysis not comprehensive. Hence, more advanced technologies are needed to cover a wider scope of cytokines along with all the polymorphisms that might be potentially associated with FMF pathogenesis. Although assessing FMF-associated symptoms and complications, namely, amyloidosis in children is quite exigent, such studies seem to be crucial to allow an early detection and management of FMF. Finally, it would be interesting to perform comparative profiles across different populations since cytokine production and genetic variations depend on patient demographics and environmental factors.

In terms of therapeutics, anti-cytokine therapy seems promising in pediatric patients resistant/intolerant to colchicine with the main limitations to its administration being its high cost and patient’s compliance. The most common anti-cytokine therapy presented in the literature was IL-1 biologics, mainly anakinra and canakinumab. Nonetheless, the studies performed were associated with some limitations especially the retrospective nature of the studies, the small sample size, and the short-term follow-up. Moreover, studies did not precisely demonstrate the effect of cytokine inhibitors alone since discontinuing colchicine treatment was extremely challenging. Admittedly, other cytokines, such as TNF-α and IL-6, were targeted as potential therapeutic agents; however, their efficiency was less significant than IL-1 biologics. Therefore, based on the provided data, future studies should include a more representative heterogenous sample with longer follow-up durations and a wider scope of biologics. Other cytokine therapies should be tested including anti-IL-17 and anti-IL-18 which are approved for use in other inflammatory conditions such as psoriasis and NLRC4/XIAP deficiencies respectively (142, 143). In accordance, designing combinational therapeutics could offer new solutions for refractory FMF patients, especially those with cytokine genetic variations associated with FMF onset and severity.

## Key points

• Exploring cytokine profile and signature in FMF provides new insights into the pathways and mechanisms involved in the disease pathogenesis.
• Cytokine studies in pediatric FMF are challenging yet crucial to design early and accurate diagnostic and therapeutic strategies that could improve patients’ quality of life.
• Anti-cytokine therapy, mainly IL-1 inhibitor, represents a safe and efficient approach to treat FMF children that are resistant/intolerant to colchicine.
• Future studies and clinical trials investigating the effect of anti-cytokine therapy in FMF should include a more representative heterogenous sample with longer follow-up durations and a wider scope of biologics (anti-IL-17, anti-IL-18, etc.).
• Designing combinational therapeutics could offer new solutions for refractory FMF patients, especially those with cytokine genetic variations associated with FMF onset and severity.
